# Immunomodulatory Effect of Polysaccharides from the Mushroom-Forming Basidiomycete *Gymnopilus imperialis* (Agaricomycetes, Basidiomycota)

**DOI:** 10.3390/ph15101179

**Published:** 2022-09-23

**Authors:** Lhaís A. Caldas, Patricia D. Santos, Elaine R. Carbonero, Marisa Ionta, Marta Miyazawa, Ester S. Caixeta, Antonio M. Fregnan, Bianca Barros Nóbrega, Maria Carolina B. Di Medeiros, Nelson Menolli, Douglas M. M. Soares, Cassius V. Stevani, Patricia Sartorelli

**Affiliations:** 1 Instituto de Ciências Ambientais, Químicas e Farmacêuticas, Universidade Federal de São Paulo, Diadema 09972-270, SP, Brazil; 2 Instituto de Química, Universidade Federal de Catalão, Catalão 75705-220, GO, Brazil; 3 Instituto de Ciências Biomédicas, Universidade Federal de Alfenas, Alfenas 37130-001, MG, Brazil; 4 Faculdade de Odontologia, Universidade Federal de Alfenas, Alfenas 37130-001, MG, Brazil; 5 Departamento de Química Fundamental, Instituto de Química, Universidade de São Paulo, São Paulo 05508-070, SP, Brazil; 6 Medicine Departament, University Center of Planalto Central Aparecido dos Santos, Brasilia 72445-020, DF, Brazil; 7 IFungiLab, Subárea de Biologia (SAB), Departamento de Ciências da Natureza e Matemática (DCM), Campus São Paulo (SPO), Instituto Federal de Educação, Ciência e Tecnologia de São Paulo, São Paulo 01109-010, SP, Brazil

**Keywords:** Atlantic Rainforest, basidiomycete, *Gymnopilus imperialis*, β-glucan, heterogalactan, nitric oxide, gene expression

## Abstract

*Gymnopilus* consists of a widely distributed genus of basidiomycetes, especially in tropical regions of the world, such as Japan, Australia, Paraguay, and Brazil. This genus biosynthesizes interesting bioactive compounds, such as sesquiterpenoids, oligoisoprenoids, styrylpyrones, and lectins. In the present study, the aqueous extract of the basidiomata of *Gymnopilus imperialis* (Basidiomycota, Agaricomycetes, Agaricales, Hymenogastraceae) was obtained by using the accelerated solvent extraction (ASE) technique, followed by the precipitation of polysaccharide fraction with ethanol. Further purification by freeze-thawing processes, Fehling solution precipitation, and membrane dialysis with different pore sizes yield three main polysaccharide fractions (*Gi*-MRSW, *Gi*-PFME, and *Gi*-SFME). According to monosaccharide composition and ^13^C-NMR data, the *Gi*-MRSW and *Gi*-SFME fractions showed to be composed mainly of β-glucans and *Gi*-PFME by a heterogalactan. Moreover, the immunomodulatory potential of *Gi*-MRSW was evaluated using RAW 264.7 murine macrophage as a study model. The nitric oxide production was significantly increased in treated samples, and the expression of inducible nitric oxide synthase (iNOS) showed that the fraction *Gi*-MRSW from *G*. *imperialis* induces the M1 polarization phenotype.

## 1. Introduction

The genus *Gymnopilus* P. Karst. (Hymenogastraceae, Agaricales) consists of an important genus of Basidiomycetes widely distributed worldwide, especially in tropical and subtropical regions [1]. Nowadays, approximately 250 species of *Gymnopilus* have been described in the literature [2], and they share similar morphological characteristics such as basidiomata brightly colored, pileus fibrillose-squamulose, lamellae adnexed to decurrent, central stipe with annulus, and spore-print fulvo-ferrigineous [3].

Some of the described species from this genus received extensive chemical and biological investigation, mainly due to their putative hallucinogenic properties caused by the psychoactive alkaloids psilocin and psilocybin [4,5]. However, most of the species remain unexploited in terms of their bioactive compounds [6]. In the Americas, some of the commonly reported species of the genus *Gymnopilus*, based on recent molecular phylogenetic studies, are *G. aeruginosus* (Peck) Singer, *G. imperialis* (Speg.) Singer, *G. junonius* (Fr.) P.D. Orton (= *G. spectabilis sensu auct.*), *G. lepidotus* Hesler, *G. luteus* (Peck) Hesler, *G. speciosissimus* Y. Lamoureux, Malloch and Thorn, *G. subspectabilis* Hesler, *G. underwoodii* (Peck) Murrill, *G. validipes* (Peck) Hesler, *G. ventricosus* (Earle) Hesler, and *G. voitkii* Malloch and Thorn [1,7,8] Among them, *G. junonius* (= *G. spectabilis sensu auct.*), popularly known as “the big laughter mushroom”, is probably the most studied species in terms of chemical constituents, mainly composed by the acetylenic compounds known as gymnopilins [9,10], although different specimens involved in the *G. junonius* complex are usually misidentified around the world [7].

Obtaining substances with antimicrobial, anticancer, immunosuppressive, immunomodulatory, antiviral, cytotoxic, and antiparasitic activities is commonly studied in plants, but basidiomycetes is still a little explored field [11,12]. Most of these activities are attributed to low-molecular-weight substances such as terpenoids, steroids, acetylenic compounds, styrylpyrones, alkaloids, and a variety of oligoisoprenoids [4,5,13,14,15,16]. However, primary metabolites such as glycoproteins and glycoconjugates also play an important and unexplored role as bioactive compounds from basidiomycetes [17]. Lectins, for example, are a group of proteins that bind to carbohydrates and glycoconjugates involved in cellular signaling of pathogenic responses, immune system interactions, and cell differentiation [18,19,20]. It has been demonstrated that lectins can act as antitumor agents due to their immunomodulatory effects [18]. Likewise, polysaccharides, macromolecules mainly composed of glucose (also named glucans), display immunomodulatory activity and represent an important constituent of the fungi cell wall [21]. Glucans are classified into α-glucan and β-glucan according to the type of glycosidic linkage and carbons involved in the bound of the glucose units. In basidiomycetes, most of the α-glucans are water-insoluble and microfibrillar, characteristic of structural polysaccharides, composed of glycogen in 1,4- and 1,6- linkages [22]. On the other hand, β-glucans present structural variability, in which glucans are bound mainly through 1,3- and 1,6- linkages [21]. It has been suggested that the wide variability of linkages and branches of β-glucans is an important key to immunomodulation, considering that the size of the molecule and its chemical complexity affects immune response [23].

The modulation of the immune system (suppression or stimulation) consists of an essential feature in preventing and controlling diseases. Some researchers have shown that fungal β-glucans can stimulate the production of macrophages and mammalian defense cells and enhance their phagocytic capacity, consequently secreting inflammatory mediators, such as tumor necrosis factor-α (TNF-α), interleukins (IL-1 β and IL-6), and nitric oxide (NO) [23,24,25].

In eukaryotic organisms, the immunologic response can occur through the release of nitric oxide, an important signaling molecule of inflammatory processes, resulting in cytoprotective effects [26]. The generation of NO is highly dependent on macrophages that induce the expression of mRNAs encoding inducible nitric oxide synthases (iNOS). iNOS catalyzes the conversion of *L*-arginine into *L*-citrulin and NO through hydroxylation and oxidation reactions consuming NADPH and molecular oxygen [26]. Polysaccharides from basidiomycetes can also exhibit anticancer properties, with direct effects on tumor activity and tumor growth [27]. This activity is especially beneficial when combined with other anticancer drugs [11]. There is evidence that polysaccharides activate macrophages, monocytes, neutrophils, natural killer cells, dendritic cells, and chemical messengers, such as interleukins, interferons, and colony-stimulating factors, which release complementary responses [11,28,29].

In this study, we evaluated the in vitro immunomodulatory potential of extracts containing polysaccharides from basidiomata of the mushroom-forming basidiomycete *G. imperialis*. For this, RAW 264.7 murine macrophages were treated with assayed extracts, and iNOS expression levels and NO production were determined. Furthermore, the gene expression profile of inflammatory modulators, such as IL10, IL6, and TNF-*α,* was also evaluated to verify the macrophage phenotype induced by treatment.

## 2. Results and Discussion

### 2.1. Taxonomy

The identity of the wild mushrooms used in this study was confirmed as *Gymnopilus imperialis* by morphological and phylogenetic studies. A phylogenetic tree (Figure S1—Supplementary Material), including the ITS sequence generated in this study, along with other sequences of many species of *Gymnopilus* worldwide, showed our Brazilian collection clustered in a well-supported clade (95% bootstrap support) with other sequences of *G. imperialis* from Brazil, Paraguay, Puerto Rico, and Costa Rica.

### 2.2. Characterization of the Polysaccharide Fractions

**Figure 1:**
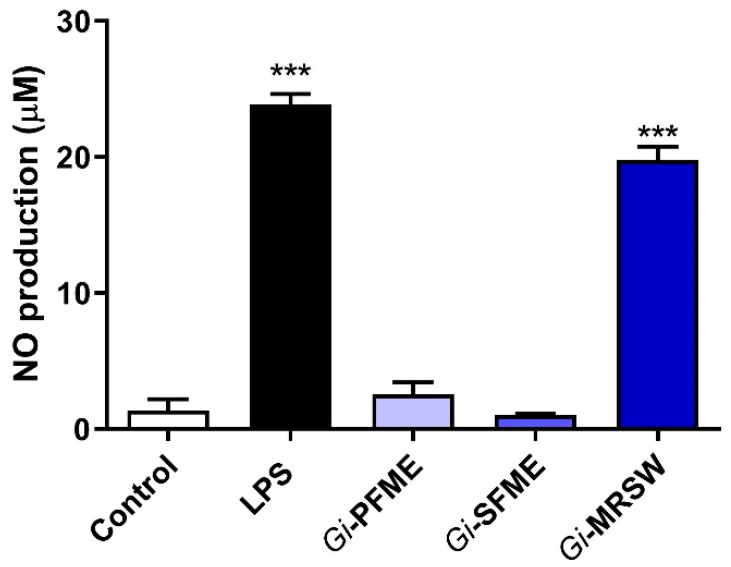
NO production determined based on the amount of nitrite by the Griess reaction. RAW264.7 cells were treated with LPS (200 ng/mL) or polysaccharides (*Gi*-PFME, *Gi*-SFME, and *Gi*-MRSW) at 40 µg/mL for 24 h. The culture medium was collected and analyzed for nitrite levels. *** *p* < 0.001 according to ANOVA followed by a Dunnett’s multiple comparison post-test.

Aqueous extract from the basidiomata of *G. imperialis* was fractionated in a series of precipitation and molecular-weight exclusion steps to obtain enriched fractions in polysaccharides (Figure 1) and to determine their monosaccharide composition and immunomodulatory potential. For purification, an aliquot of the lyophilized extract (3.6 g) was submitted to ethanol precipitation (*Gi*-EP 1.2 g), followed by dialysis with 12–14 kDa cut-off furnishing the crude polysaccharide fraction (*Gi*-W 780.0 mg). After dissolution with distillate water, the *Gi*-W was fractionated with a freezing/thawing process, which furnished cold-water-soluble (*Gi*-SW 670.0 mg) and insoluble (*Gi*-IW 86.0 mg) fractions that were separated by centrifugation. *Gi*-SW was dialyzed in a molecular exclusion dialysis membrane of 1000 kDa cut-off, originating a retained fraction (*Gi*-MRSW 66.8 mg) and an eluted fraction in the membrane (*Gi*-MESW 400.0 mg). Then, an aliquot of the *Gi*-MESW fraction (176.0 mg) was treated with Fehling’s solution, also known as a method of precipitation through coordination metals, which is based on the knowledge that polysaccharides are able to form coordination precipitates with metallic compounds, such as an insoluble Cu^2+^ complex [30]. After centrifugation, two fractions were obtained, a Fehling’s supernatant (*Gi*-SFME 92.0 mg) and a Fehling’s precipitate fraction (*Gi*-PFME 60.4 mg).

**Figure 2:**
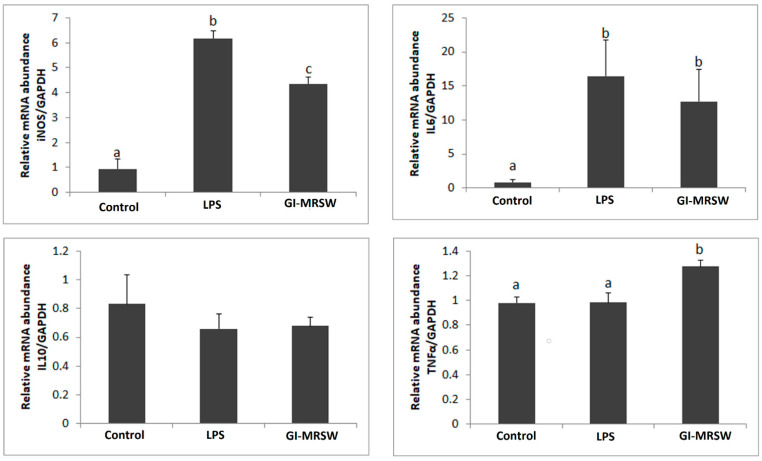
Relative mRNA abundance determined by qPCR after 24 h of the treatment using *GAPDH* as a housekeeping gene. RAW 264.7 cells were treated with LPS (200 ng/mL) or fraction *Gi*-MRSW at 40 µg/mL for 24 h. Different letters represent groups with significant differences in gene expressions pattern.

From this fractionation procedure, three main cold-water-soluble fractions (*Gi*-MRSW, *Gi*-PFME, and *Gi*-SFME) were obtained and submitted to GC-MS analysis (as alditol acetates, after derivatization) (Table 1) and to ^13^C NMR spectroscopy (Figure 2). The analyses of monosaccharide composition showed high glucose contents for the *Gi*-MRSW and *Gi*-FSME fractions, suggesting that they were composed mainly of glucans. On the other hand, the *Gi*-FPME fraction was shown to consist mainly of galactose, indicating the presence of a heterogalactan containing lateral chains formed by mannose and fucose (Table 1).

Analysis of ^13^C NMR corroborates the presence of β-glucans as the main signals observed in the spectra of *Gi*-MRSW and *Gi*-FSME fractions that are characteristic of this class of compounds [31]. Signals from the *Gi*-FPME fraction correspond to a heterogalactan, commonly found in macrofungi, which contains a main chain of α-Gal*p* (1→6)-linked, partially substituted in O-2 by non-reducing ends of β-Man*p* and α-Fuc*p* [32]. The signals observed in the anomeric region of the ^13^C NMR spectrum of the *Gi*-FPME fraction (Figure 2) refer to the C-1 of the non-reducing ends of β-Man*p* (δ 104.36) and α-Fuc*p* (δ 104.14), and the main chain units consisting of α-Gal*p* 6-*O*- (δ 100.89) and 2,6-di-*O*-substituted (δ 101.48 and 101.18). The glycosidic linkage of type 1→6 of the α-Gal*p* units of the main chain was confirmed by the signal in δ 69.85, while the substitutions in O-2 of these units by non-reducing terminals of α-Fuc*p* and β-Man*p* were confirmed by the signs in δ 80.54 and 79.82, respectively [32]. Although the ^13^C NMR spectra of *Gi*-MRSW (Figure 2) and *Gi*-FSME (Figure 2) fractions suggest the majority presence of β-glucans in these fractions, they have distinct structures. The ^13^C NMR data of *Gi*-MRSW showed similarities with schyzophillan-type glucan, consisting of a main chain of β-Glc*p*(1→3)- connected units and containing branches in O-6 by non-reducing terminals of β-Glc*p*, on average one branching every three units of the main chain [31]. The glycosidic linkages of the glucan were shown by the presence of 3-*O*-substituted signals at δ 86.61, 86.23, and 85.99, and O-substituted-CH_2_-6 signal at δ 68.46. On the other hand, the ^13^C NMR spectrum of the *Gi*-FSME fraction showed characteristic signals of another type of β-glucan that has been isolated from macrofungi, as described for *Cantharellus* *cibarius* [33], which has a higher proportion of (1→6) linkages when compared to (1→3), aside containing a main chain distinct from that reported for *Gi*-MRSW.

Glucan-type polysaccharides from many edible and medicinal basidiomycetes are vastly described in the literature, sorted by their monosaccharide composition and immunomodulatory potential. There are reports on the immunomodulatory polysaccharides CVPn and CVPa from the edible and medicinal mushroom *Trametes* *versicolor* (= *Coriolus versicolor*) [34]; the polysaccharide content of the edible *Paxillus involutus* [27]; the glucan-type water-soluble polysaccharide, FVP1, isolated from the edible *Flammulina velutipes* [24]; as well as the polysaccharides F1, and F2, isolated from the edible *Schizophyllum commune*, whose glucose composition is approx. 88% of the monosaccharide composition [23,24]. Literature data suggest that polysaccharides composed of glucose, galactose, and mannose are more likely to be recognized by cell membrane receptors, such as toll-like receptors, mannose receptors, and complement receptors in immunologic responses [35,36]. This fact may result in the expression of cytokines and iNOS related to immunomodulation [37]. Aside from their relatively simple monosaccharide units, some features such as the number of branches, the anomeric configuration of glucose units, the molecule conformation, the molecular weight, and the type of glycosidic linkage can significantly alter the biological activities of these carbohydrates [38].

### 2.3. Immunomodulatory Potential

Fungal polysaccharides are able to trigger innate and adaptive immune responses against pathogens and antigens. Innate immunity develops non-specific responses right after recognizing one specific foreign body and initiates immunomodulatory responses through macrophages, dendritic cells, cytokines, interleukins, interferons, and others [39]. In innate immunology, polysaccharides are known to be able to produce immune responses by activating macrophages, leading to the production of nitric oxide (NO), upregulation of tumor necrosis factor (TNF-α), interleukin 6 (IL-6), and interleukin 12 (IL-12) [24]. In this way, it was observed that fraction *Gi*-MRSW significantly stimulated NO production through the Griess reaction (Figure 1), whose potency was similar to LPS, a well-known macrophage activation factor [40]. NO is crucial intracellular signaling regarding host defense and activation of macrophages in immune responses.

Moreover, the iNOS relative mRNA abundance was significantly higher in samples treated with fraction *Gi*-MRSW for 24 h than in untreated groups. These findings corroborate those results obtained by the Griess assay. Furthermore, the gene expression profile of some inflammatory modulators was also evaluated to verify whether *Gi*-MRSW induces the M1 or M2 phenotype of RAW 264.7 cells. The M1 macrophages are closely related to the defense of the organism against external pathogens or tumor cells by releasing cytotoxic and inflammatory mediators, such as NO, TNF-α, IL-6, and IL1-β; whereas M2 macrophages typically produce anti-inflammatory mediators, including IL-10 and TGF-β [41] leading to immunosuppression and tumor progression. By contrast, the immunosuppression in the tumor microenvironment is associated with infiltrating tumor-associated macrophages (TAMs) and their M2 phenotype, which is characterized by IL-10 ^high^, TGF-α ^high^, IL-6 ^low^, and TNF-α ^low^ production [42]. In the present study, we found upregulation of TNF-α and IL-6 in samples treated with fraction *Gi*-MRSW compared to untreated groups. LPS increased IL-6 mRNA levels in the experimental conditions but did not modify TNF-α relative mRNA abundance (Figure 2). It may be possible that changes in TNF-α mRNA levels have occurred before the analyzed period. It has been reported that the releasing peak of TNF-α occurs 24 h after LPS stimulation in THP-1, PBMC, and monocytes; by contrast, IL-6 secretion was observed after 24 h of treatment with LPS [43]. Therefore, our findings indicate that fraction *Gi*-MRSW displays an immunostimulatory activity on murine macrophages and effectively induces the M1 polarization phenotype.

The observed immunomodulatory potential of polysaccharides is in agreement with previous studies with the polysaccharide LVF-I from the edible mushroom *Lactifluus volemus* (= *Lactarius volemus*), which induced macrophage activation through upregulation of NO, IL-6, and TNF-α [37]. Moreover, similar results were also observed for polysaccharides from *Tremella fuciformis* [44], *Lentinula edodes* [45], *Grifola frondosa* [46], and *Auricularia auricula-judae* [47].

## 3. Materials and Methods

### 3.1. Mushroom Collection and Taxonomy

The wild mushrooms studied were collected in April 2016 in an Atlantic Rainforest remnant in Southeastern Brazil (Iporanga, São Paulo: S24°33.51′, W48°43.54′, elevation 500–530 m). The morphological data used for taxonomy were based on notations from fresh and dried basidiomata and compared to previous descriptions [48,49]. The remaining basidiomata were deposited at Herbarium SP at the ‘Instituto de Pesquisas Ambientais’ (IPA, Saão Paulo, SP, Brazil) under the number SP513080.

The molecular analysis to support the identification and infer the phylogenetic positioning of the specimen sampled was based on ITS1-5.8S-ITS2 (ITS) sequences of nuclear rDNA [50] from both the basidiomata and the pure culture. Genomic DNA extraction, amplification by PCR (polymerase chain reaction), purification, and sequencing were conducted as previously described in the literature [8] and using the universal ITS5F and ITS4R primer set [51]. The consensus ITS sequences from both the basidiomata and the pure culture were generated in Geneious 7.0.6 and deposited at GenBank under the following accession codes: OP297374 (basidiomata) and OP297375 (pure culture). A single dataset was generated from alignment conducted in MAFFT 7 (http://mafft.cbrc.jp/alignment/server/ (accessed on 25 July 2022)) [52] with auto strategy, and including 91 sequences of *Gymnopilus* species, most of them previously generated [1,7,8] and recovered from a BLASTn search (https://blast.ncbi.nlm.nih.gov/Blast.cgi (accessed on 25 July 2022)) against the NCBI GenBank database based on the newly generated sequence in this study. Additionally, an ITS sequence from an authentic *G. imperialis* from Paraguay [48] was kindly provided by M.G. Campi for comparison and then deposited at GenBank under the accession code OP094030. Two sequences of *Galerina allospora* A.H. Sm. and Singer (AJ585511 and AJ585452) were chosen as outgroups based on literature [53]. The alignment was visually examined and manually corrected using Geneious 7.0.6 [54], and then a maximum likelihood (ML) analysis was conducted in RAxML 8.2.9 using the GTR+GAMMA+I model with a rapid bootstrap analysis with 1000 replicates and search for the best-scoring ML tree [55].

### 3.2. Extraction of the Compounds

Wild mushrooms of *G. imperialis* were lyophilized and grounded in a mortar using a pestle yielding 86 g of dried powder. The extraction process was performed by using ASE 350 equipment (Accelerated Solvent Extractor, Dionex ASE 350, Thermo Fisher Scientific, Waltham, MA, USA) [20,56]. Briefly, 84.0 g of material was mixed with 56.0 g of diatomaceous earth (Sigma, CA, USA), and the mixture was loaded onto the 66 mL extraction cell between two 0.45 μm cellulose membrane filters. Distilled water was used as a solvent for the extraction under 1500 psi at 80 °C. Heat time was set to 5 min, with 5 min static time, 60% flush volume, 120 s purge time, and three static cycles. One hundred milliliters of solution yielded by the extraction were concentrated under reduced pressure and then lyophilized (Labconco FreeZone 2.5 L Benchtop, EUA).

### 3.3. Fractionation of the Compounds

**Figure 3:**
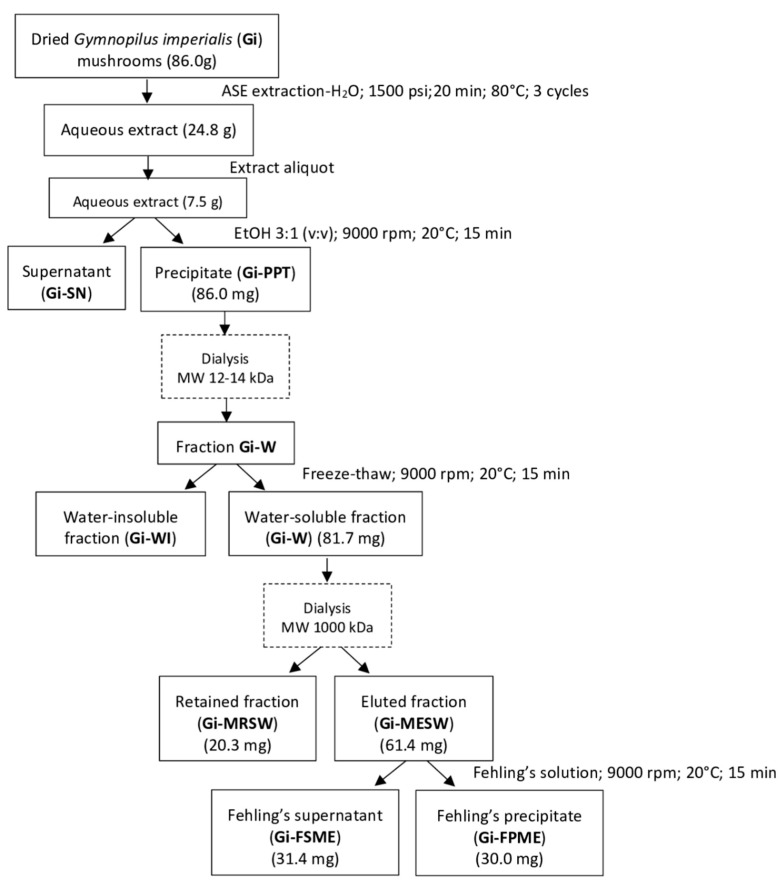
The general procedure of polysaccharide purification from the basidiomata of *Gymnopilus imperialis*.

The lyophilized aqueous extract of the basidiomata of *G. imperialis* was submitted to ethanol precipitation (3:1, *v*/*v*). Polysaccharide fractions were recovered through centrifugation (5000 rpm, 20 min, 15 °C) dialysis using a 12–14 kDa cellulose membrane (Sigma-Aldrich^®^, St. Louis, MO, USA) submerged in a container filled with distilled water over 36 h to remove low-molecular-weight carbohydrates. After that, the retained fraction was lyophilized. The polysaccharide fraction *Gi*-EtOH ppt (16.3 g) was fractioned following the freeze-thawing methodology [57]. Previously, the fraction was solubilized in distilled water and submitted to this process. Insoluble precipitates in cold water were separated from the other fraction through centrifugation. After that, all originated fractions were lyophilized. In the following step, soluble fractions were submitted to dialysis with a Biotech CE Tubing 1000 kDa *M_r_* cut-off membrane (Spectra/Por^®^) [30]. At this point, polysaccharide fractions were submitted to a Fehling solution treatment [58]. After 12 h of refrigeration, insoluble copper complexes were separated through centrifugation (5000 rpm, 20 min, 15 °C). The obtained fractions, supernatant, and precipitate were neutralized using acetic acid and dialyzed in a 12–14 kDa cut-off membrane for 48 h. After dialysis, the fractions were submitted to filtration with DOWEX 50 WXB resin (Sigma-Aldrich^®^) for copper ion removal (Figure 3).

### 3.4. Monosaccharide Composition

Analysis of the monosaccharide composition was carried out according to the following: total acidic hydrolysis, reduction, and acetylation of the hydrolysis products. Briefly, the resulting fraction was then analyzed by GC-MS [59]. Monosaccharide components of the polysaccharides were identified, and their ratios were determined following hydrolysis with 2M trifluoroacetic acid (TFA) for 8 h at 100 °C, and their conversion to alditol acetates by successive NaBH_4_ reduction, and acetylation with Ac_2_O-pyridine (1:1, *v/v*) for 12 h at room temperature [59]. The resulting alditol acetates were analyzed by GC-MS using an Agilent 7820A gas chromatograph coupled to a mass spectrometer Agilent 5975E, both equipped with silica capillary column DB-225-MS (0.25 mm × 30 m × 0.25 μm), using electron impact as the ionization source and a quadrupole analyzer, fitted with split/splitless capillary inlet system, an Agilent G4513A autosampler. Injections of 1 µL were made in the splitless mode at an injection temperature of 250 °C and detector at 280 °C. The column oven temperature was initially held at 50 °C for 1 min. Then, it was programmed at 40 °C/min to 220 °C or 210 °C (constant temperature) for quantitative analysis of alditol acetates and partially *O*-methylated alditol acetates, respectively. Helium was the carrier gas at a flow rate of 1 mL/min. Electron impact (EI) analysis was performed with the ionization energy set at 70 eV.

### 3.5. NMR Measurement

The ^13^C NMR spectra were acquired on a spectrometer Bruker Avance III in an 11.7 Tesla (500 MHz for hydrogen frequency), using temperature 70 °C, TSP-*d_4_* as an internal reference, solvent D_2_O or Me_2_SO-*d_6_* and the broadband direct observe detection probehead except for ^13^C. The parameters were acquisition time (AQ = 0.52 s), spectral width (SWH = 31,250 Hz), relaxation delay (d1 = 0.1 s), and number of scans (ns = 4 K). All data were analyzed using *TopSpin* software, and the chemical shifts were signaled in ppm, calibrated against the TMS signal, internal reference.

### 3.6. Cell Line and Culture Conditions

RAW murine macrophage cell line (RAW 264.7) was used in the present study. The cells were grown in Dulbecco’s Modified Eagle’s Medium (DMEM/F12, Sigma, CA, USA) supplemented with 10% of fetal bovine serum (SFB, Vitrocell, Campinas, Brazil). Cells were grown in a humidified atmosphere with 95% air and 5% CO_2_ at 37 °C (CO_2_ incubator, Thermo Fisher, Waltham, MA, USA).

### 3.7. Measurement of Nitric Oxide (NO) Production

**Figure 4:**
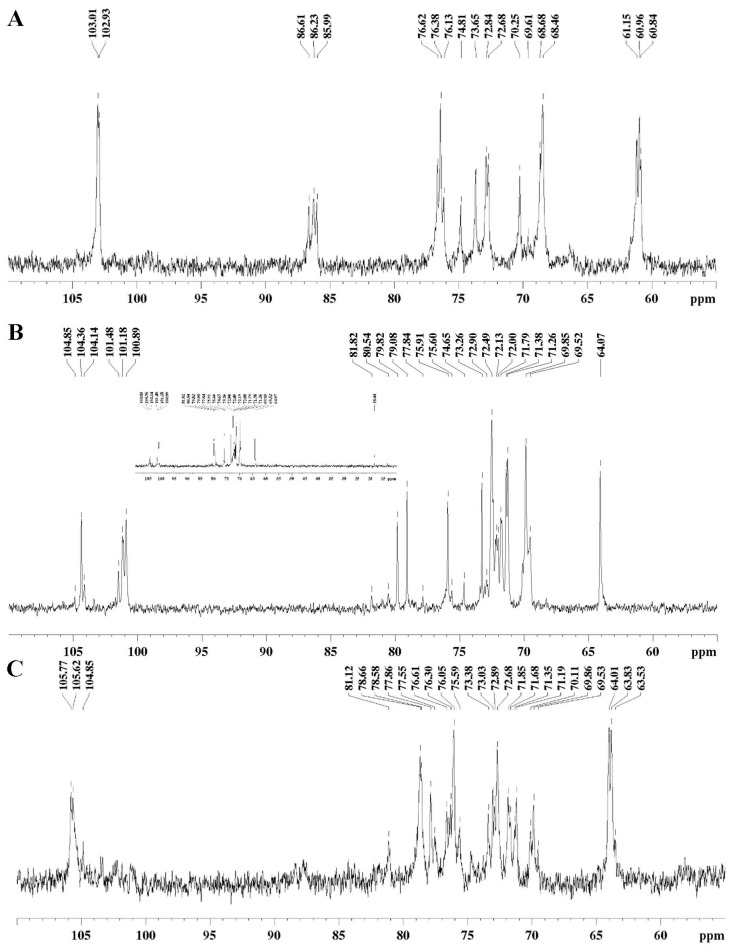
^13^C NMR spectra of the *Gi*-MRSW (**A**), *Gi*-FPME (**B**), and *Gi*-FSME (**C**) fractions obtained at 70 °C from the basidiomata of *Gymnopilus imperialis*.

The cells were seeded into a 96-well plate at 5 × 10^4^ cells/well. After 24 h, the cells were stimulated with lipopolysaccharide (LPS) at 200 ng/mL or fractions (1–10) at 40 µg/mL for 24 h. Polysaccharide extracts were used in different concentrations (5, 10, 20 or 40 µg/mL) for 24 h. NO levels were determined by measuring nitrite levels in the culture media using Griess reagent assay [60]. Briefly, 50 µL of supernatant were mixed with the same volume of Griess reagent, and the resultant mixture was then incubated for 10 min. The absorbance at 570 nm was then recorded, and NaNO_2_ was used as the standard to calculate (0, 1.56, 3.12, 12.5, 25, 50, and 100 µL) the nitrite concentration [61]. Results are represented as mean ± standard deviation (SD) from three independent experiments performed in quadruplicate (Figure 4). Significant differences were evaluated using a one-way analysis of variance (ANOVA) followed by a Dunnett’s multiple comparison post-test using GraphPad Prism^®^ ( version 8.0, San Diego, CA, USA ).

### 3.8. Transcript Level Expression of Target Genes

Total RNA from the treated and untreated RAW 264.7 was extracted using the RNeasy^®^ Micro kit (Qiagen, Mississauga, ON, Canada), according to the manufacturer’s instructions, and eluted in 30 μL of RNAse-free water. RNA concentrations were measured by spectrophotometer using a NanoDrop^®^ ND 1000 (Thermo Scientific, Wilmington, DE, USA), and 1 µg of total RNA was incubated with DNase I (1 U/µg, Invitrogen, São Paulo, SP, Brazil) to eliminate possible contamination with genomic DNA, and then subjected to reverse transcription using random primers and the High-Capacity cDNA Reverse Transcription Kit^®^ (Applied Biosystems, São Paulo, SP, Brazil), according to the manufacturer’s instructions. The reagents were incubated at 25 °C for 10 min, 37 °C for 120 min, and finally, 85 °C for 5 min to inactivate the enzyme. Expression of the target genes (IL6, IL10, iNOS, and TNF-α) was investigated by real-time PCR with an ABI 7500 thermocycler using Power Sybr Green PCR Master Mix (Applied Biosystems) using specific primers (Table 2). The final volume was 25 μL, and the genes were amplified using the following conditions: 95 °C for 10 min (1 cycle), denaturation at 95 °C for 10 s, and annealing and extension at 60 °C for 1 min (40 cycles). To select the most stable housekeeping gene, β-actin (ACTB), glyceraldehyde-3-phosphate dehydrogenase (GAPDH), and 18S ribosomal RNA (18S rRNA) amplification profiles were compared using the geNorm applet for Microsoft Excel (medgen.ugent.be/genorm) [62]. The relative abundance of each target gene was calculated using the ΔΔCt method with efficiency correction and a control sample for calibration [63]. The average efficiency values for each gene were calculated through the amplification profile of each sample using the LinRegPCR program [64]. Each sample was run in triplicate, and non-template control was included. The data are presented as mean ± standard error of the mean (SEM) from four independent experiments.

### 3.9. Statistical Analysis

The results were tested for significance using a one-way analysis of variance (ANOVA) followed by Dunnet’s post-test using GraphPad Prism^®^. The values were expressed as mean ± SD from three experiments.

## 4. Conclusions

*Gymnopilus imperialis,* whose identity was assigned based on phylogenetic and morphologic studies, is a basidiomycete that accumulates bioactive polysaccharides. In the present study, the aqueous extract was obtained from dried basidiomata of *G. imperialis*, which was fractioned by several steps yielding three main polysaccharide fractions (*Gi*-MRSW, *Gi*-FSME, and *Gi*-FPME). Among these cold-water-soluble fractions, only the *Gi*-MRSW fraction significantly stimulated NO production, demonstrating its immunomodulatory ability on RAW 264.7 murine macrophage. Moreover, it was found that this treatment also significantly upregulates iNOS expression. Taken together, the data show that the *Gi*-MRSW fraction from *G*. *imperialis* displays immune-enhancing activity on murine macrophages through induction of the M1 polarization phenotype.

## Figures and Tables

**Table 1:** Monosaccharide compositions of the cold-water-soluble fractions obtained from the basidiomata of *Gymnopilus imperialis*.

Fractions	Monosaccharides (%)			
	Fucose	Mannose	Galactose	Glucose
*Gi*-MRSW	-	14.1	21.9	64.0
*Gi*-PFME	7.1	30.7	58.8	3.4
*Gi*-SFME	-	22.8	11.4	65.8

**Table 2:** Information of specific primers used for amplification in real-time PCR.

Gene	Sequence	Reference
*IL6*	F 5′-CTGCAAGAGACTTCCATCCAG -3′	NM_031168.2
	R 5′-AGTGGTATAGACAGGTCTGTTGG -3′	
*IL10*	F 5′-CTTACTGACTGGCATGAGGATCA -3′	NM_010548.2
	R 5′-GCAGCTCTAGGAGCATGTGG -3′	
*iNOS*	F 5′-GTTCTCAGCCCAACAATACAAGA -3′	NM_010927.4
	R 5′-GTGGACGGGTCGATGTCAC -3′	
*TNFα*	F 5′-CTGAACTTCGGGGTGATCGG -3′	NM_013693.3
	R 5′-GGCTTGTCACTCGAATTTTGAGA -3′	
*GAPDH*	F 5′-AACGACCCCTTCATTGAC -3′	NM_001289726.1
	R 5′-TCCACGACATACTCAGCA -3′	

## Data Availability

Data is contained within the article and supplementary material.

## Supplementary Materials

The following supporting information can be downloaded at: https://www.mdpi.com/article/10.3390/ph15101179/s1, Figure S1: Best tree of Maximum Likelihood analysis of Gymnopilus species with *Galerina allospora* as outgroup. Bootstrap values > 70% are shown near the node branches. The terminal names in bold represent the sequences of G. imperialis generated for this work. The small figure linked to the *G. imperialis* clade is the photo of the fresh mushrooms used in this study. Original names of each terminal were maintained as available in GenBank but the bigger and bold names on the left represent the putative current name for each clade.
