# Supplementary figures and images for: Immunomodulatory Effect of Polysaccharides from the Mushroom-Forming Basidiomycete Gymnopilus imperialis (Agaricomycetes, Basidiomycota)
Source: Pharmaceuticals (Basel). 2022 Sep 23;15(10):1179. doi: 10.3390/ph15101179 (PMC9611870; doi:10.3390/ph15101179)

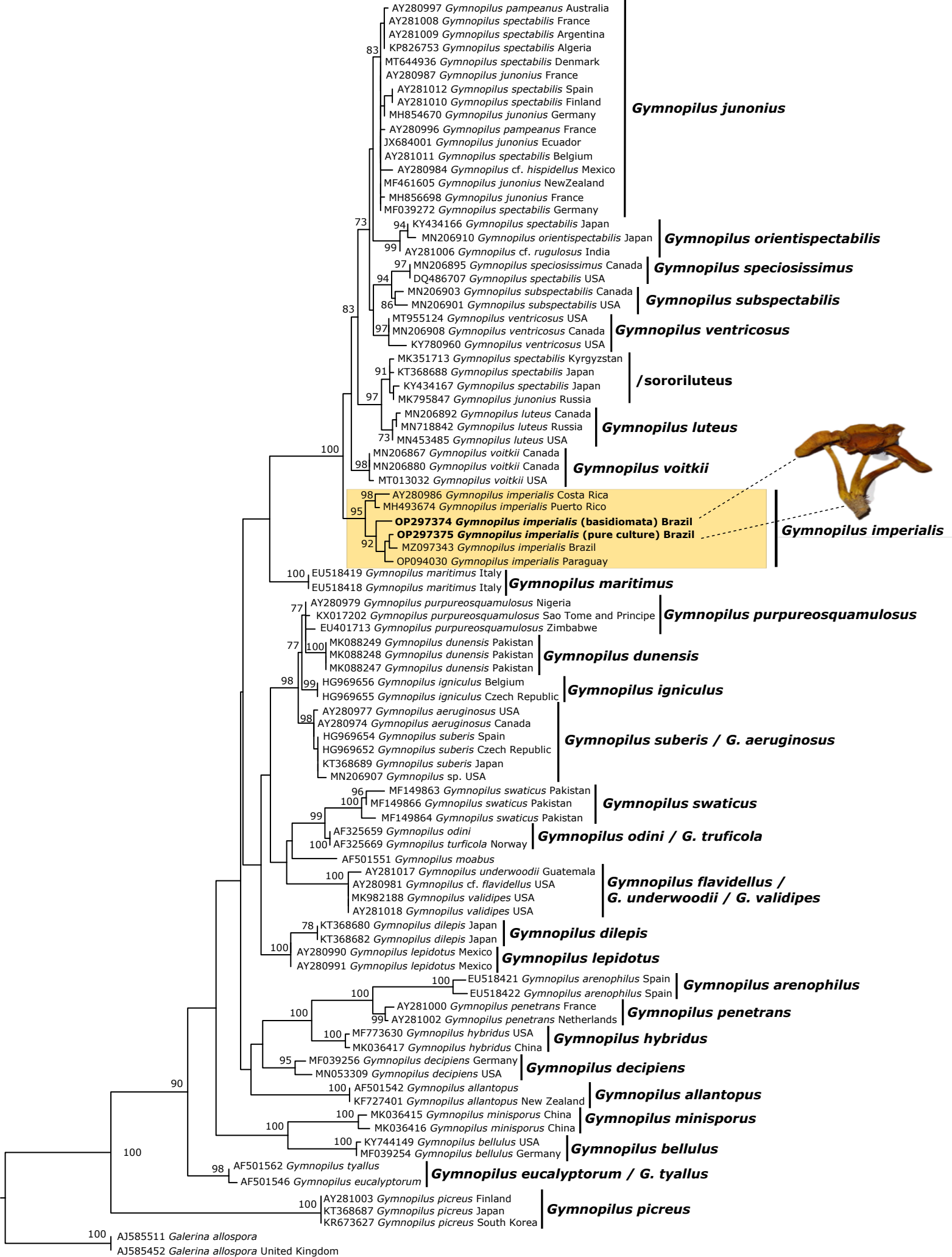

Supplement: Supplementary file 1 [file pharmaceuticals-15-01179-s001.zip › pharmaceuticals-1914662-supplementary-figure S1.pdf]
